# Solution Structure of a Phytocystatin from *Ananas comosus* and Its Molecular Interaction with Papain

**DOI:** 10.1371/journal.pone.0047865

**Published:** 2012-11-06

**Authors:** Deli Irene, Tse-Yu Chung, Bo-Jiun Chen, Ting-Hang Liu, Feng-Yin Li, Jason T. C. Tzen, Cheng-I Wang, Chia-Lin Chyan

**Affiliations:** 1 Department of Chemistry, National Dong Hwa University, Hualien, Taiwan, Republic of China; 2 Graduate Institute of Biotechnology, National Chung Hsing University, Taichung, Taiwan, Republic of China; 3 Department of Chemistry, National Chung Hsing University, Taichung, Taiwan, Republic of China; 4 Singapore Immunology Network, Biomedical Sciences Institutes, Agency for Science, Technology and Research (A*STAR), Singapore; National Institute for Medical Research, Medical Research Council London, United Kingdom

## Abstract

The structure of a recombinant pineapple cystatin (AcCYS) was determined by NMR with the RMSD of backbone and heavy atoms of twenty lowest energy structures of 0.56 and 1.11 Å, respectively. It reveals an unstructured N-terminal extension and a compact inhibitory domain comprising a four-stranded antiparallel β-sheet wrapped around a central α-helix. The three structural motifs (G^45^, Q^89^XVXG, and W^120^) putatively responsible for the interaction with papain-like proteases are located in one side of AcCYS. Significant chemical shift perturbations in two loop regions, residues 45 to 48 (GIYD) and residues 89 to 91 (QVV), of AcCYS strongly suggest their involvement in the binding to papain, consistent with studies on other members of the cystatin family. However, the highly conserved W120 appears not to be involved in the binding with papain as no chemical shift perturbation was observed. Chemical shift index analysis further indicates that the length of the α-helix is shortened upon association with papain. Collectively, our data suggest that AcCYS undergoes local secondary structural rearrangements when papain is brought into close contact. A molecular model of AcCYS/papain complex is proposed to illustrate the interaction between AcCYS and papain, indicating a complete blockade of the catalytic triad by AcCYS.

## Introduction

Cystatins, the natural inhibitors of cysteine proteases are important regulatory proteins found in mammals, plants, and insects [1], [2], [3]. They are involved in various biological and pathological processes, such as tumor invasion, inflammation, antigen processing, dystrophy, and metastasis [4]. The cystatin superfamily in higher animal is generally classified into three subfamilies according to their size and the presence of internal disulfide bonds [5], [6], [7]. Members of family 1, termed stefins are small intracellular proteins of about 100 residues with no disulfide bond. Cystatins of family 2 are extracellular proteins of about 120 residues with at least two internal disulfide bonds. Members of family 3, termed kininogens, are relatively large blood-circulated glycoproteins composed of several family 2-like cystatin domains.

Previous studies have shown that the family 1 and 2 cystatin homologs share a common fold comprising an antiparallel β-sheet wrapped around a central α-helix [8], [9], [10], [11], [12]. The co-crystal structure of human stefin B/papain complex revealed the interaction of a wedge-shaped edge of the inhibitor with the enzyme active-site cleft [12]. Three structural elements essentially responsible for the specific binding of cystatins to the active-site cleft of papain-like cysteine proteases are a conserved Gly at the N-terminus, a QxVxG motif situated in the β-hairpin loop between the second and the third strand, and an aromatic residue on the β-hairpin loop between the fourth and the fifth strand [13], [14], [15]. The parasite chagasins, cystatin-like proteins were newly found to have a similar recognition pattern with cysteine proteases [16], [17], [18], [19], [20]. Although chagasins strongly inhibit cysteine proteases, the three inhibitory loops of chagasins show low sequence homology to other cystatins.

Several plant cystatin genes were cloned and their deduced proteins were found homologous to animal cystatins in the past decade [21], [22], [23], [24], [25], [26], [27], [28]. As a consequence of their sequence uniqueness, these plant cystatin-like proteins are classified as a new subfamily termed phytocystatins. Phytocystatins have been proposed to have several possible functions, including regulating the activity of endogenous cysteine proteases during different physiological processes such as seed maturation and germination, as well as responding to biotic and abiotic stresses [27], [29], [30], [31]. Phytocystatins are also implicated in programmed cell death by modulating cysteine protease activities in the regulation of protein turnover [32], [33]. They may also be involved in defense mechanisms to protect plants from the invasion of pathogens or the attack by pests [34]. Therefore, phytocystatins appear to function as inhibitors of both endogenous and exogenous cysteine proteases and may have potential applications in agriculture and medicine.

Sequence analysis suggests that phytocystatins also possess the three structural elements essential for the interaction with papain-like proteases. In addition, phytocystatins contain a signature motif LARFAVxEHN in the α-helix forming sequence but lack disulfide bonds and potential glycosylation sites [6]. Although most of the phytocystatins are small proteins with molecular weight of 12–16 kDa, some phytocystatins possess an N-terminal signal sequence and/or a C-terminal extension that involves in the inhibition of a second family of cysteine proteases, legumain proteases [35]. Furthermore, several multicystatins that contain multiple copies of cystatin domains have also been identified [24], [36], [37], [38].

A *c*DNA fragment encoding a phytocystatin of pineapple (*Ananas comosus* L.) stem was successfully cloned in our previous study [22]. To explore the potential applications of this pineapple cystatin (AcCYS), we aimed to unravel its inhibitory action at atomic level in this study. The recombinant AcCYS, consisting of 135 residues without any cysteine residue, was expressed in *Escherichia coli* and purified to apparent homogeneity. NMR techniques were employed to determine the structure of AcCYS and to characterize its interaction with papain. A docking model of AcCYS/papain was also proposed to illustrate the inhibitory action of AcCYS toward papain.

**Figure 1:**
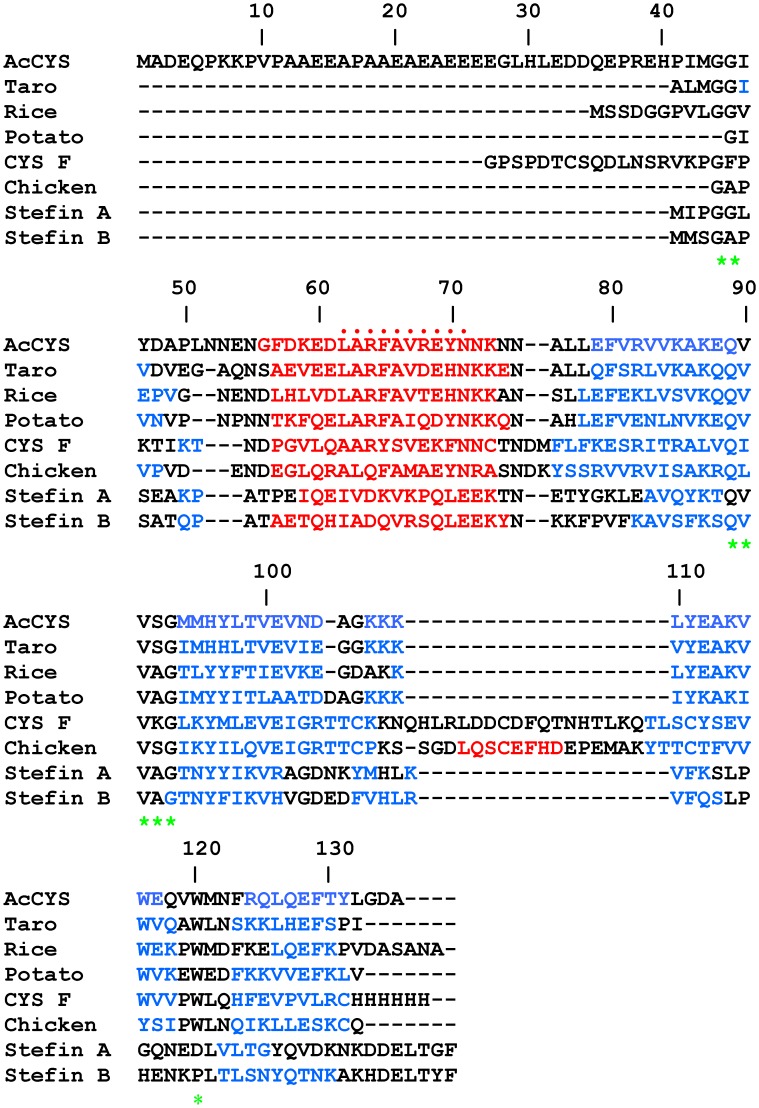
Sequence alignment of AcCYS to the other cystatins. The sequence of AcCYS is compared with taro, rice, potato, human cystatin F, chicken egg white cystatin, human stefin A, and human stefin B. The characteristic motifs for putative interaction with target cysteine protease are marked by green asterisks. The unique motif, LARFAVxExN of phytocystatins is denoted by the red dots on top of the sequence. The α-helix and β-sheet regions are colored in red and blue, respectively.

## Materials and Methods

### Expression and Purification of Full Length and Truncated AcCYS

The *AcCYS* gene encoding a cysteine protease inhibitor, cystatin was cloned from pineapple (*Ananas comosus*) stem [22]. The gene was then subcloned into a modified pET28a expression vector. A truncated form of *AcCYS* gene (*AcCYS-DL*) containing the coding gene of residue 28–135 was amplified by PCR, then subcloned into a modified pET28a expression vector. Both constructs were verified by DNA sequencing, and then transformed into *E. coli* BL21 (DE3) host for protein expression. After expression and cell disruption, the recombinant AcCYS (residue 1–135) and AcCYS_DL (residue 28–135) proteins were purified by anion exchange (DEAE, GE) followed by size exclusive (Superdex G75, GE) chromatography. ^13^C, ^15^N-labeled AcCYS and AcCYS_DL were overexpressed in a modified M9 medium containing 1 g/l ^15^NH_4_Cl and 2 g/l glucose (^13^C_6_-glucose) as the sole ^15^N and ^13^C sources. The purified recombinant AcCYS and AcCYS_DL ran as a single band on SDS-PAGE. Protein concentrations of AcCYS and AcCYS_DL were determined by UV spectrometer using the extinction coefficient ε_280nm_, 18450 M^−1^ cm^−1^.

**Table 1:** Structural statistics of 20 lowest-energy structures of AcCYS.

A. Restraints	
Intra-residue NOEs (| i−j | = 0 )	446
Sequential NOEs (| i−j | = 1 )	417
Medium range NOEs (2≤ | i−j | ≤5 )	213
Long range NOEs (| i−j |>5)	553
Total NOEs	1629
Dihedral angle restraints	
φ	55
ψ	55
Hydrogen bond restraints	43
**B. Ensemble Statistics Analysis**	
*RMSD values (residues 40–135, Å)*	
Backbone atoms	0.56±0.12
Heavy atoms	1.11±0.16
*Statistics from Ramachandran plot (%)*	
Residues in most favored regions	75.4
Residues in additional allowed regions	23.3
Residues in generously allowed regions	1.3
Residues in disallowed regions	0.0

### Preparation of NMR Samples and NMR Spectroscopy

The AcCYS and AcCYS_DL proteins were dissolved in a solution containing 20 mM KCl, 0.02% NaN_3_, and 10% (v/v) D_2_O, and the pH was adjusted to 6.5. The final concentrations of the AcCYS were ranged from 0.7 to 1.1 mM for structure determination. AcCYS_DL/papain complex was prepared as follows: 1 mg ^13^C, ^15^N-labeled AcCYS_DL was dissolved into 1 ml solution containing 90% H_2_O/10% D_2_O, 20 mM KCl, 0.02% NaN_3_, at pH 6.5. The appropriate amount of unlabelled papain stock solution (0.5 mM) was added dropwise into the AcCYS_DL solution with gentle mixing to ensure AcCYS_DL/papain complex formation. The solution was then concentrated by centricon-30 ultrafiltration apparatus (Millipore Inc) to a final concentration of about 0.25 mM. The final NMR sample solutions were transferred to 5 mm Shigemi NMR tubes (Shigemi Co., Tokyo, Japan).

**Figure 2:**
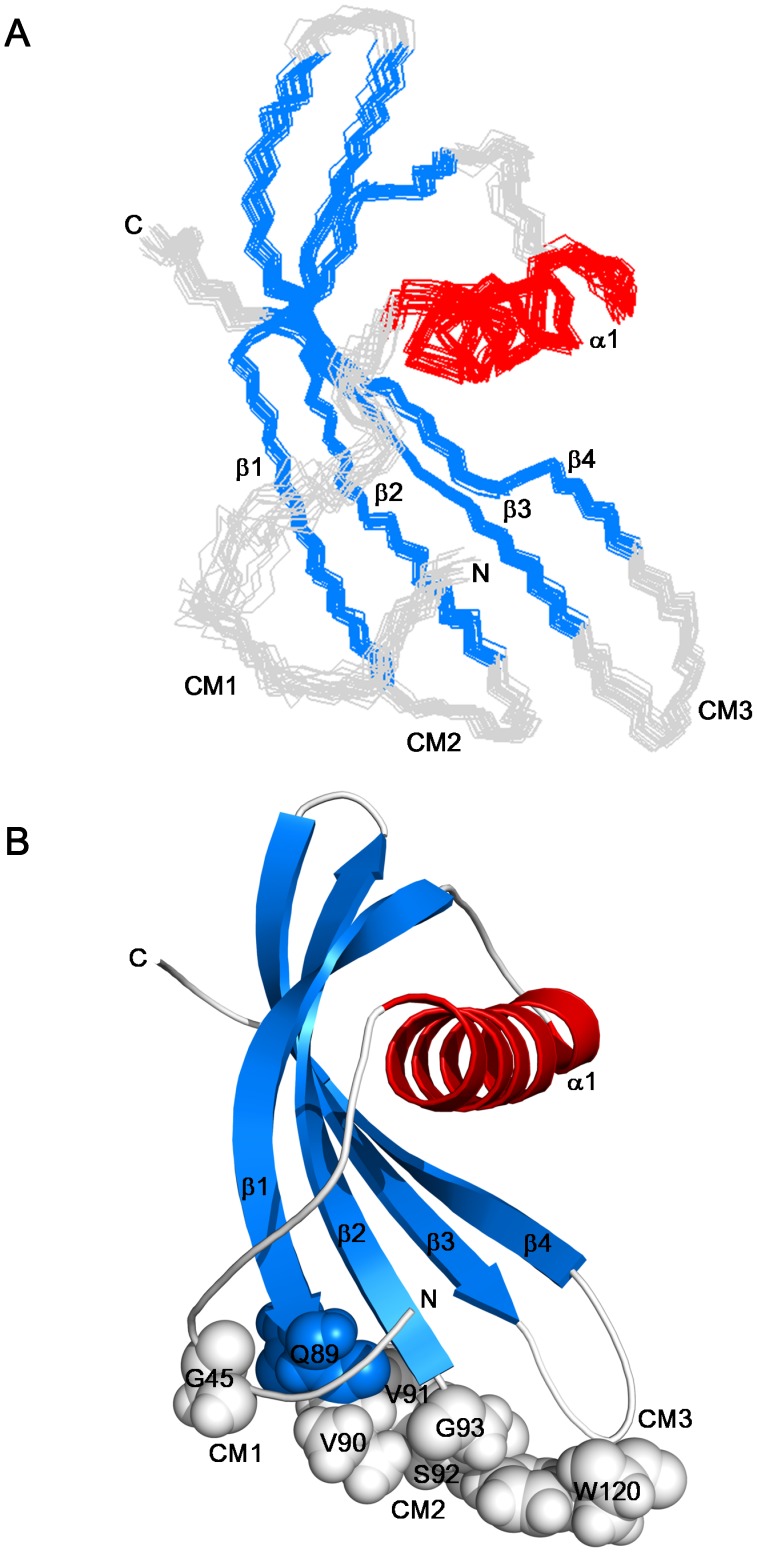
Solution structure of AcCYS. (**A**) The ensemble of 20 best structures of the inhibitory domain of AcCYS (res 41 to 135) is superimposed. The α-helical, β-sheet, and loop region is color coded in red, green, and grey respectively. (**B**) Ribbon representation of the inhibitory domain of AcCYS structure shows a αβ roll structure made up of one α-helix and four anti-parallel β-strands, β_1_–β_4_. Three regions which contain the highly conserved motifs are labeled as CM1–3. Side chains of these highly conserved residues including, G45, Q^89^VVSG, and W120 are shown in spheres. These diagrams were generated using the structural visualization program PYMOL.

All NMR data were recorded at 303 K on Bruker AVANCE-600 spectrometer equipped with xyz-gradient TXI probe in National Dong Hwa University. All the spectra were processed by TOPSPIN and analyzed by AURELIA on Linux workstations. Proton chemical shifts were referenced to DSS, and ^13^C and ^15^N chemical shifts were calibrated indirectly using the absolute frequency ratios.

**Figure 3:**
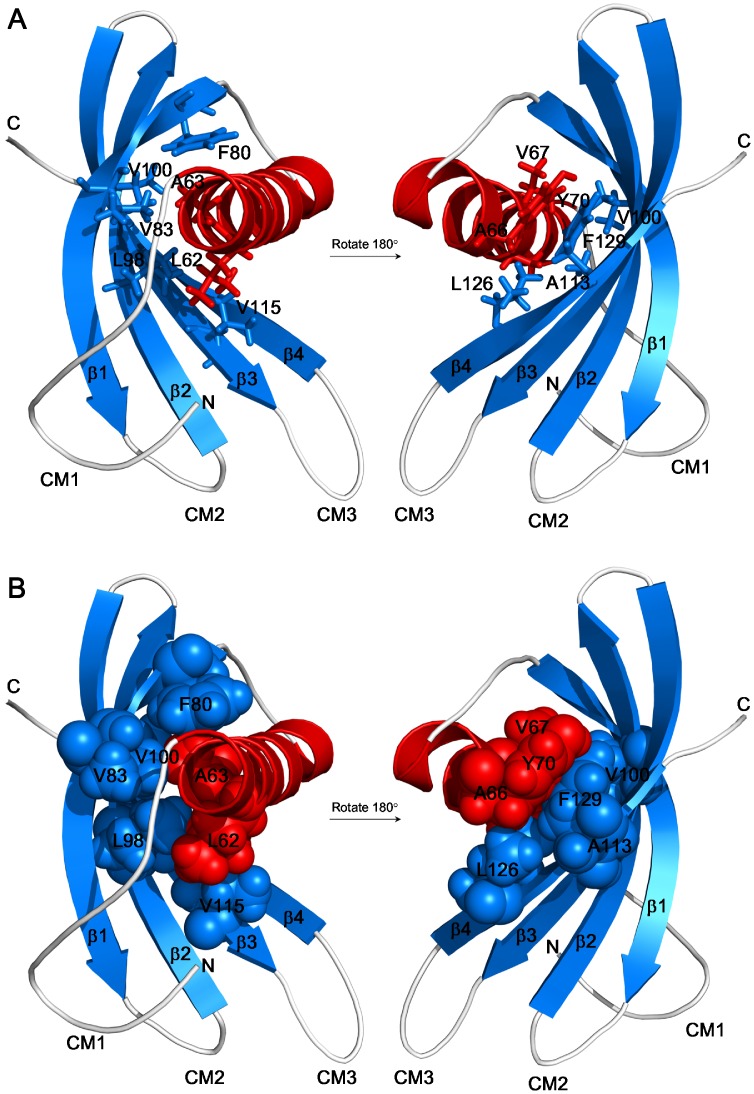
Hydrophobic clusters of AcCYS on the interface of α-helix and β-sheet. Hydrophobic side chains that participate in stabilizing the compact structure of AcCYS are labeled and shown in sticks (**A**) and in spheres (**B**).

### Resonance Assignments and Structure Calculation of Free AcCYS

Resonance assignments of free AcCYS were obtained using a series of double- and triple-resonance NMR experiments and have been reported in our previous study [39]. To assign NOE-based distance restraints, 3D ^13^C-separated and ^15^N-separated NOESY-HSQC spectra were recorded on uniformly ^13^C and ^15^N-labeled samples respectively. The NOE crosspeaks were picked and quantified by peak-picking algorithm in Aurelia. The NOE peak intensities were converted into upper distance bounds using CALIB module in the torsional angle dynamics program CYANA [40]. The backbone dihedral angles (φ and ψ) restraints were derived by chemical shifts of ^1^H^α^, ^13^C^α^, ^13^C^β^, ^13^CO, and ^15^N nuclei using the program TALOS [41]. Hydrogen bond restraints were assigned between slowly exchanging amide protons and their respective carbonyl acceptors deduced from the NOE data in combination with the secondary structure information predicted from CSI [42]. Initial structures were generated by the automated module, CANDID/NOEASSIGN in CYANA. These NOE assignments were carefully confirmed and erroneous ones corrected through examination of spectra. Additional NOE were then added manually before recalculation of structures by CYANA. The final 20 conformers with the lowest target function values were selected and further refined by the restrained simulated annealing and energy minimization algorithms in CNS 1.3 [43], [44]. Graphical visualization and analyses of the structures were carried out with the programs MOLMOL [45] and PyMOL (DeLano Scientific). The geometric and stereo-chemical quality of the ensemble of structures was validated by PROCHECK-NMR [46].

**Figure 4:**
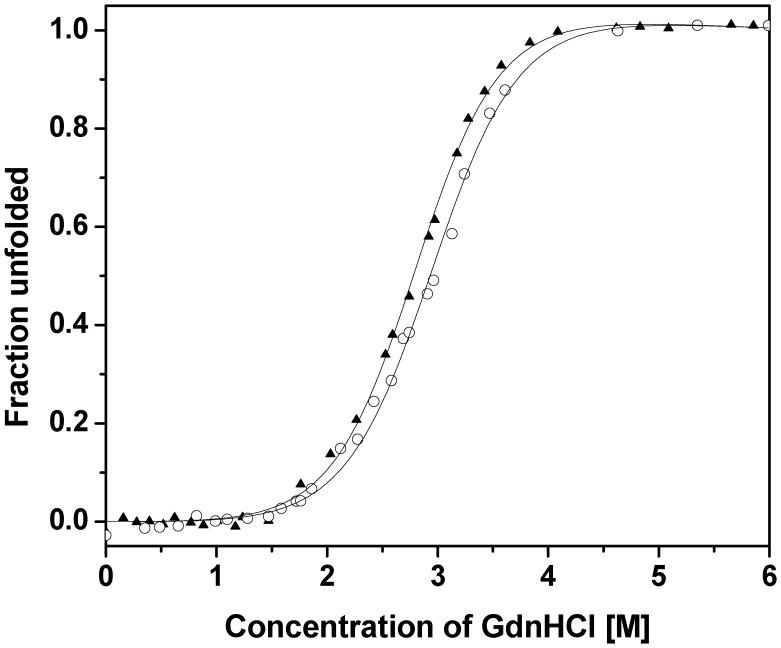
Equilibrium unfolding of AcCYS and AcCYS_DL. The GdnHCl-induced unfolding curves of AcCYS (▴) and AcCYS_DL (○) were monitored by CD at the wavelength of 222 nm. The transition curve was quantified and the corresponding value of 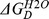 for AcCYS and AcCYS_DL is 4.4, and 4.5 Kcal/mol, respectively.

### Circular Dichroism (CD) Spectroscopy and Thermodynamic Stability Measurements

CD spectra were recorded on a Jasco J-715 circular dichroism spectrometer. CD spectra were collected using a cylindrical quartz cuvette with a 1 mm path-length. Measurements were made for the purified recombinant AcCYS and AcCYS-DL at a concentration of 10 µM. Each CD spectrum was averaged over 16 scans and corrected for the appropriate buffer baseline. All spectra are presented as the molar CD absorption coefficient (Δε_M_). The contents of secondary structures were analyzed by the Dichroweb program [47], [48].

**Figure 5:**
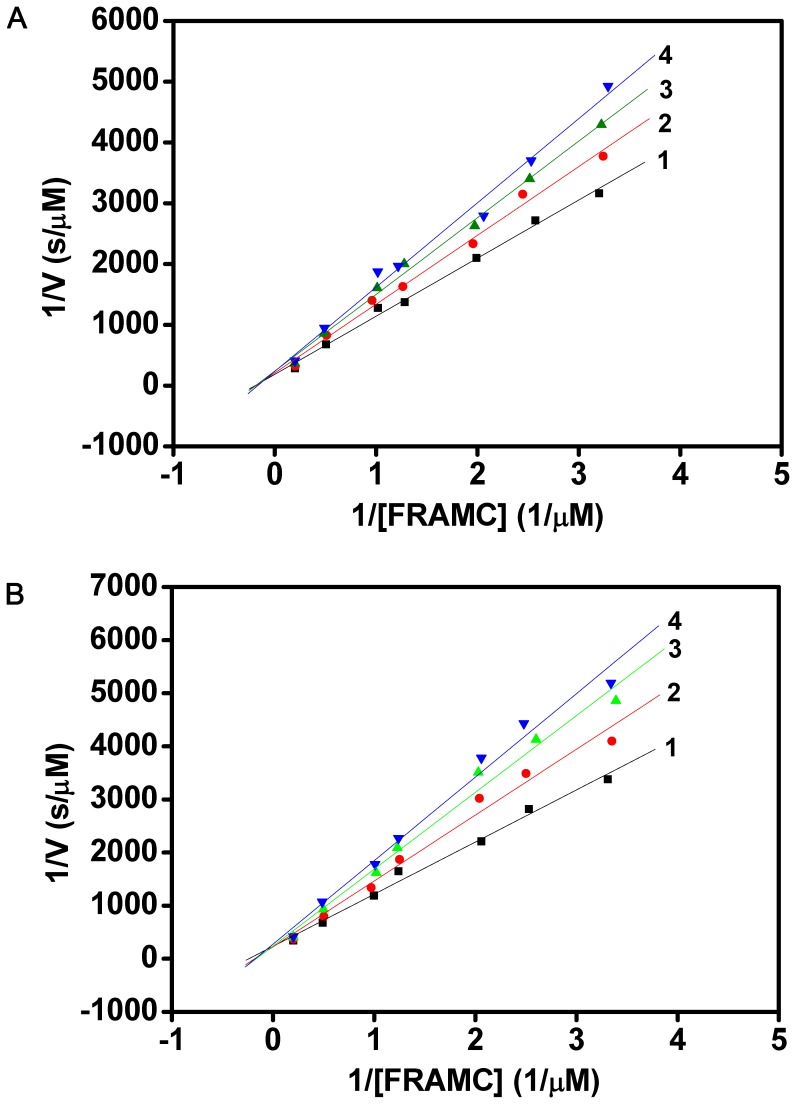
The Lineweaver-Burk plots for the inhibition of papain by AcCYS and AcCYS-DL. The inhibition of papain by AcCYS and AcCYS-DL is shown in (**A**) and (**B**) respectively. The initial rates of cleavage of a fluorogenic substrate, Z-Phe-Arg-7-amido-4-methylcoumarin (FRAMC) hydrochloride by papain were obtained spectrofluorometrically with excitation and emission wavelengths at 346 and 450 nm, respectively. Line 1 represents papain activity (2 nM) in the absence of inhibitor. Line 2–4 show the enzyme activity in the presence of 40, 60, 80 pM cystatin, respectively. Both AcCYS and AcCYS-DL strongly inhibited papain with inhibitory constant K_I_ of 2.0±0.2×10^−10^ M and 1.4±0.1×10^−10^ M, respectively.

The thermodynamic stability measurements for the unfolding of AcCYS and AcCYS_DL against denaturant GdnHCl was performed and calculated using a classical two-state model as shown in equation 1 [49].
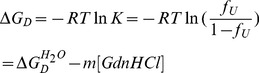
(1)where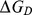 and 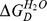 represent the free energy of unfolding of proteins in the presence and absence of denaturant respectively; f_U_ is the fraction of unfolded state; *m* is a measure of the dependence of free energy on GdnHCl concentration. The fraction of the unfolded state (f_U_) can be expressed as equation 2.



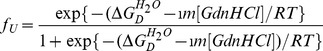
(2)Experimental data can be fitted according to this equation by the program Origin 6.0 (Microcal Software Inc.).

**Figure 6:**
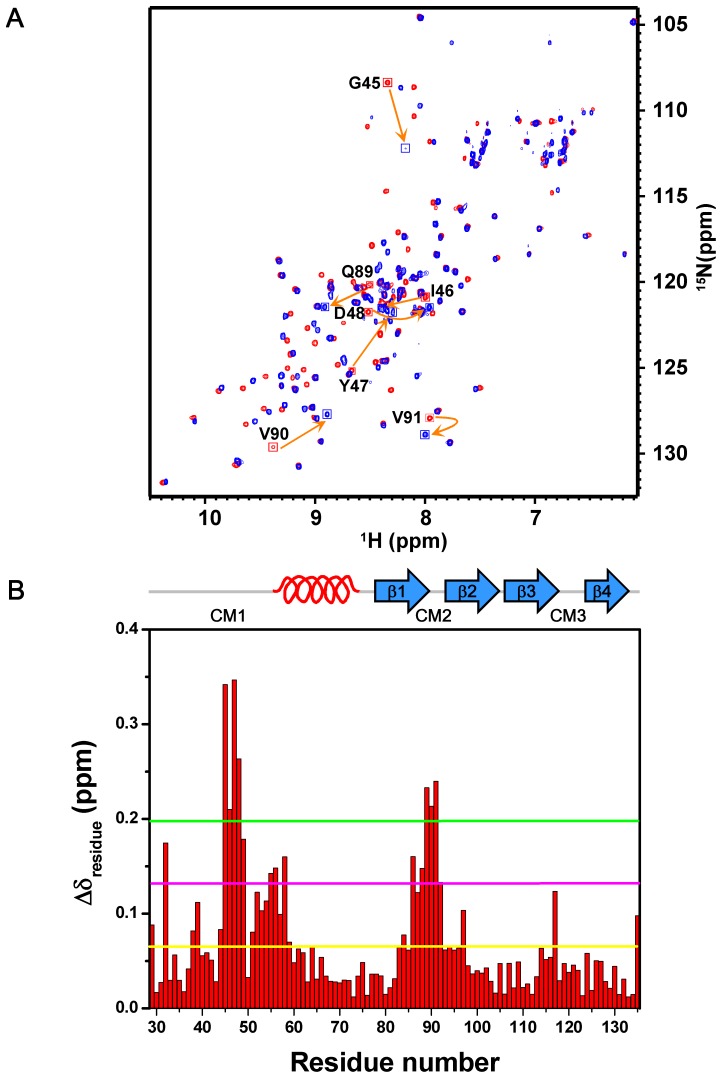
Complex formation of AcCYS_DL and papain. (**A**) ^15^N-^1^H HSQC spectra of AcCYS-DL alone (red) and in association with papain (blue). The residues with most significant chemical shift perturbation are labeled. (**B**) The overall chemical shift perturbation, Δδ_residue_ of AcCYS_DL upon association with papain was plotted against residue number. Δδ_residue_ of each residue was calculated as described in Materials and Methods. The average of Δδ_residue_ for all residues of AcCYS_DL upon association with papain was calculated and shown as the yellow horizontal solid line (0.066 ppm). The pink and green horizontal solid lines represented two and three fold averaged Δδ_residue_, respectively.

**Figure 7:**
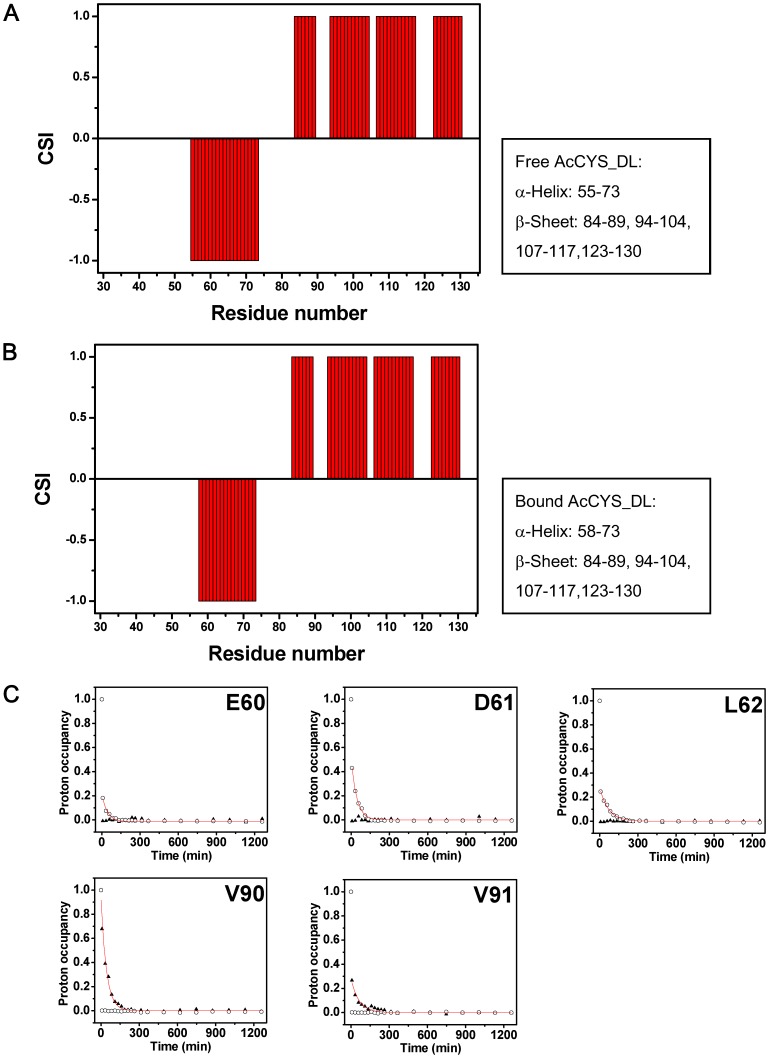
Local secondary structural rearrangements of AcCYS when associated with papain. (**A**) CSI plot of free AcCYS_DL. (**B**) CSI plot of AcCYS_DL upon association with papain. (**C**) H/D exchange of amide protons of V90, V91, E60, D61, and L62 in the bound form (▴) and in the free state of AcCYS DL (○).

### Inhibitory Activity of AcCYS and AcCYS-DL to Cysteine Proteases

The inhibition constants (K_I_) for AcCYS and AcCYS_DL to a cysteine protease, papain (Sigma, Missouri, USA) were determined. A fluorogenic substrate, Z-Phe-Arg-7-amido-4-methylcoumarin hydrochloride, FRAMC (Calbiochem, Darmstadt, Germany) was used for the assay. The initial rates of cleavage of FRAMC by papain were obtained spectrofluorometrically. The inhibitory activity was assayed in a 100 mM sodium phosphate buffer, pH 6.3 with 10 mM EDTA, 400 mM NaCl, and 2 mM dithiothreitol. Substrate concentration was ranged from 0.3 to 5 µM and substrate hydrolysis never exceeded 5%. The fluorescence was measured in an Amico-Bowman Series 2 spectrofluorometer with excitation and emission wavelengths at 346 and 450 nm, respectively. The kinetic data were processed by Lineweaver-Burk plot analysis.

**Figure 8:**
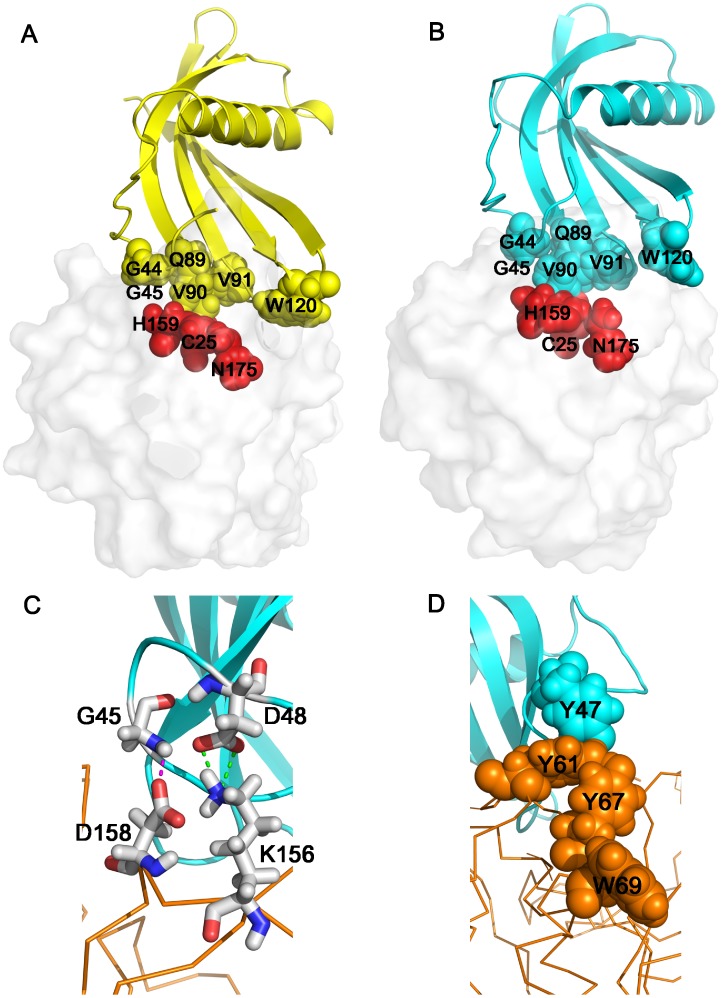
Structural model of AcCYS/papain complex. (**A**) The complex structure of AcCYS/papain was simulated by ZDOCK. AcCYS is shown in yellow ribbons. The binding site of AcCYS is shown in yellow spheres. The surface of papain is shown in light grey. The active site of papain including C25, H159, and N175 residues are shown in red spheres. (**B**) Complex structure of AcCYS/papain determined by restrained molecular dynamics simulations. AcCYS is shown in light blue ribbons. The G44–G45 and Q89–V91 of AcCYS shown in spheres completely block the active site of papain. The first turn of α-helix, residue 56–58 collapses. (**C**) AcCYS/papain complex stabilized by intermolecular forces. The backbone of papain is shown in yellow line. An intermolecular H-bond between G45 amide proton of AcCYS and the side chain carboxyl of D158 of papain and an intermolecular salt bridge between the carboxyl of D48 of AcCYS and K156 of papain are formed in the simulated complex structure (shown in sticks). (**D**) A new hydrophobic cluster between AcCYS and papain. The side chain of Y47 of AcCYS (shown in light blue spheres) as well as Y61, Y66, and W69 residues of papain (shown in yellow spheres) form an intermolecular aromatic cluster.

### NMR Spectroscopy and Resonance Assignments of AcCYS_DL and AcCYS_DL when Associated with Papain

Backbone resonance assignments of free AcCYS_DL were obtained by correlating intra- and inter-residue through-bond connectivities of ^13^C^α^, ^13^C^β^, and ^13^C' in a series of standard 2D and 3D NMR spectra including ^15^N-^1^H HSQC, HNCO, HN(CA)CO, HNCA, HN(CO)CA, CBCANH, and CBCA(CO)NH. Assignments of H^α^, H^β^ were obtained by ^15^N-TOCSY-HSQC, HBHA(CACBCO)NH, and HCCH-TOCSY spectra. Resonance assignments of ^15^N, ^13^C-labeled AcCYS_DL when associated with unlabeled papain were first performed by comparing chemical shifts with those in free AcCYS_DL. Ambiguous chemical shifts were then obtained by correlating intra- and inter-residue resonances in standard 3D NMR spectra using similar procedures as in the assignments for free AcCYS_DL. Chemical shifts were confirmed by inspection of intra-residue and sequential NOEs in ^13^C,^ 15^N-edited NOESY-HSQC spectra. Chemical shift perturbation map was used to identify the papain-binding site of AcCYS_DL. A single quantity used as normalized chemical shift perturbation of each residue (Δδ_residue_) was expressed as equation 3.
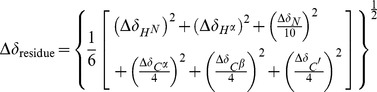
(3)Δδ_HN_, Δδ_Hα_ Δδ_N_, Δδ_Cα_ Δδ_Cβ_ and Δδ_C′_ represent the chemical shift difference of AcCYS_DL upon association with papain for nucleus H^N^, H^α^, N, C^α^, C^β^, and CO respectively. The secondary structure rearrangement of AcCYS_DL upon association with papain was predicted by CSI program.

### Computational Analysis

Bioinformatics analysis of AcCYS with other cystatin sequences from different families was performed with CLC workbench (CLC bio). Multiple sequence alignments were performed using CLUSTALW algorithm [50]. Prediction of signal peptide was performed using Target P 1.1 server [51]. The solved structure of AcCYS was analyzed and classified by CATH [52] and DALI [53] algorithms.

### Molecular Models of AcCYS in Association with Papain

Molecular modeling of AcCYS in association with papain and their docking and restrained molecular dynamic simulation were performed using Discovery Studio 2.1 platform (Accelrys, San Diego, CA). The initial structure model of AcCYS/papain complex was generated from the taro cystatin/papain crystal structure (PDB ID: 3IMA) with the taro cystatin replaced by our NMR AcCYS structure (PDB ID: 2L4V). This complex structure was then energy minimized with CHARMM force field [54]. Docking simulations of AcCYS to papain were performed by use of rigid body ZDOCK 2.1 [55]. The binding site of AcCYS and papain was defined as those residues with significant chemical shifts perturbation upon complex formation and in close contact (≤5 Å) with each other in our initial structure model. The orientation of AcCYS was obtained with 6° rotational sampling, and 54000 predictions were generated. The structure with the lowest ZRank score was selected and subsequently subject to energy minimization. The complex structure of AcCYS/papain obtained from ZDock was then further simulated with restrained molecular dynamics using a cascade protocol in Discovery Studio 2.1. The procedures in the restrained molecular dynamics simulation included minimization with steepest descent and conjugate gradient methods, followed by heating and equilibration dynamics under 300 K, and finished with production dynamics. The distance restraints used in the molecular dynamics simulation include hydrogen bonds derived from H/D hydrogen exchange and CSI data, intramolecular restraints of hydrophobic clusters in AcCYS, and intermolecular restraints derived from the dock model. The distance-dependent dielectric constant of the solvent was used in the molecular dynamics simulation. The lowest dielectric constant was 1 and the solvent dielectric constant was 80. The conformation of the AcCYS/papain complex with the lowest potential energy in the production dynamics was selected to present the complex structure of AcCYS/papain.

### Accession Numbers

^1^H, ^13^C, and ^15^N chemical shift assignments were deposited in the Biological Magnetic Resonance Data Bank (http://www.bmrb.wisc.edu) under accession code 17258. The coordinates of the final ensemble of 20 structures and the NMR restraints used for structural determination were deposited in the PDB under accession code 2L4V.

## Results and Discussion

### Sequence Alignments

The sequence of AcCYS was aligned with other selective cystatin homologs in Figure 1. Three characteristic motifs of cystatins which were believed to form three-point interaction with target cysteine protease were identified. They included a region containing one or two G residue at the N-terminal region, a QxVxG motif, and a region containing a W residue near C-terminal region. The unique motif LARFAVxExN of phytocystatins was also found in AcCYS. Furthermore, AcCYS possesses an unusual AE-rich sequence of approximately 30 residues in its N-terminus. This AE-rich sequence is unique to AcCYS as verified by extensive blast in databanks.

### Structure of Free AcCYS

The solution structures of AcCYS were calculated using CYANA 2.1. There are 568, 913, and 148 NOE distance restraints deduced from the ^15^N-separated, ^13^C-separated NOESY-HSQC, and 2D-NOESY for aromatic residues, respectively. In addition to NOE distance restraints, 43 distance restraints for hydrogen bonds and 55 backbone φ and ψ restraints were used for final calculation of the structures. An ensemble of 20 conformers with lowest restraints violations was selected for further analysis. Statistical parameters for the 20 calculated structures were listed in Table 1. Root mean square deviations of these structures were 0.56±0.12 Å for backbone atoms and 1.11±0.16 Å for all atoms (residues 40 to 135). The quality of the structural ensemble was evaluated by the program PROCHECK-NMR, in which 98.7% of the residues were in the most favored and additionally allowed regions, only 1.3% in generously allowed region, and no residues in disallowed region in the Ramachandran plot. The final refined ensembles of 20 lowest energy structures have been deposited into the protein data bank (PDB) with accession code 2L4V.

Superposition of the 20 best structures of AcCYS together with ribbon diagrams of the averaged structure was shown in Figure 2. AcCYS contains an unstructured N-terminal domain (residues 1 to 40), a loop (residues 41 to 46) and a compact inhibitory domain (residues 47 to 135). The N-terminal AE-rich sequence is located in the unstructured region. The compact inhibitory domain is classified by CATH as a αβ roll structure comprising an α-helix and a four-stranded β-sheet with the secondary structural topological arrangement of α−β_1_−β_2_−β_3_−β_4_. The α-helix, residues 56–73, is wrapped around by anti-parallel β-strands comprised of residues 79–89 (β_1_), 94–104 (β_2_), 107–117 (β_3_), and 124–131 (β_4_). The inner two β-strands, β_2_ and β_3_, are coiled smoothly, but the outer two β-strands, β_1_ and β_4_, are twisted with three β-bulges on residues V81, V84, and Q127. Together, these bulges produce a tight coiling of the β-sheet, allowing it to wrap around the helix. By DALI 3D-clustering algorithm, the inhibitory domain of AcCYS shows high structural similarity to potato (PDB entry 2W9Q) and taro (PDB entry 3IMA) cystatins with RMSD of 1.9 and 2.1 Å, respectively. However, the short N-terminal β-strand found in residues 24–25 of potato cystatin and residues 6–7 of taro cystatin is not observed in the corresponding residues 46–47 in AcCYS. Three structural motifs (G45, Q^89^XVXG, and W120) putatively responsible for the interaction with papain-like proteases are located on one side of AcCYS.

The compact globular structure of AcCYS is mainly stabilized by the hydrophobic clusters on the interface between the α-helix and the β-sheet as shown in Figure 3. The key hydrophobic residues of α-helix, including L62, A63, A66, V67, and Y70, are located on the phytocystatin-specific motif, LARFAVxExN. The structurally complementary hydrophobic residues on β-sheet, including F80, V83, L98, V100, A113, V115, L126, and F129, also show high homology to other phytocystatins.

### Secondary Structure and Bulk Thermodynamic Stability Measurements by Circular Dichroism

To assess the effects of the N-terminal extension sequence on the native structure of AcCYS, CD was used to estimate the secondary structure contents of AcCYS and its truncated form, AcCYS_DL (residue 28–135) in the far-UV region. The estimated α-helix and β-sheet contents are 14% and 31% for AcCYS, and 15% and 33% for AcCYS_DL, suggesting that the overall secondary structures of AcCYS are not affected by the removal of the N-terminal AE-rich sequence (residue 1–27). We then compared the stabilities of AcCYS and AcCYS_DL by analyzing their unfolding against denaturant, as monitored by the decrease in the secondary structure content of the proteins. The fraction of unfolded state was plotted as a function of GdnHCl concentration (Figure 4). The unfolding curves of both AcCYS and AcCYS_DL showed cooperative characteristics. The transition curve was quantified by the methods described in Materials and Methods, giving 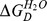
values of 4.4, and 4.5 Kcal/mol for AcCYS and AcCYS_DL, respectively. These observations indicate that the removal of N-terminal AE-rich sequence does not affect the thermodynamic stability of AcCYS against GdnHCl-unfolding.

### Inhibitory Activity of AcCYS and AcCYS-DL to Papain

A proteolytic activity assay using fluorogenic substrate FRAMC was established to investigate the effect of the N-terminal sequence in AcCYS on its inhibitory activity toward papain. The Lineweaver-Burk plots of the assays with various concentrations of inhibitors AcCYS and AcCYS-DL were shown in Figure 5A and B. The inhibition constants (K_I_) for AcCYS and AcCYS-DL to papain were determined according to the plots. Both AcCYS and AcCYS_DL strongly inhibited papain with K_I_ of 2.0±0.2×10^−10^ M and 1.4±0.1×10^−10^ M, respectively. The results indicate that the removal of the N-terminal sequence in AcCYS has slightly increased the inhibitory ability against papain.

### Recognition of AcCYS to Papain Determined by Chemical Shift Perturbation, CSI, and H/D Exchange Studies

Since the unstructured N-terminal region was readily fragmented by papain within NMR acquisition time, we therefore used AcCYS_DL to characterize the interaction. As chemical shifts are sensitive to changes in chemical environments caused either by proximity to the interaction surface or by structural readjustments, they are frequently used as atomic resolution probes to monitor the interaction between biomolecules. We herein used chemical shift perturbation to map the interaction site of AcCYS_DL to papain. Complexes of labeled AcCYS_DL with unlabeled papain were prepared and their NMR spectra were acquired. Backbone resonance assignments of AcCYS_DL in free and bound forms are completed using the procedures described in Materials and Methods and listed in (Figure S1 and Table S1). The ^15^N-HSQC of the complex of labeled AcCYS_DL with unlabeled papain was overlaid with the spectrum of free AcCYS_DL (Figure 6A). The majority of the crosspeaks of AcCYS_DL shifted upon association with papain. The normalized chemical shift deviation of each residue, Δδ_residue_ was calculated as described in Materials and Methods and plotted against residue number as shown in Figure 6B. The average Δδ_residue_ for all residues of AcCYS_DL upon association with papain was 0.066 ppm. The most perturbed residues of AcCYS_DL of which the Δδ_residue_ are larger than three folds of the averaged Δδ_residue_ were found in two conserved loop regions, residue 45 to 48 (GIYD) and residue 89 to 91 (QVV). These loops are likely to be in the proximity to the interaction surface and are predicted to play pivotal roles in the recognition to papain. However, the highly conserved W120 was found to have a chemical shift perturbation less than the average of Δδ_residue_ and therefore was likely not crucial in the binding to papain. The other strongly perturbed residues of AcCYS_DL with Δδ_residue_ within two to three folds of averaged Δδ_residue_ were found in the first turn of α-helical region (residues 55, 56 and 58) and the first strand of the β-sheet (residues 86 and 88). These residues are not located on the primary interaction surface and therefore are suspected to undergo induced fit upon binding of AcCYS_DL to papain. In order to prove the hypothesis, we used chemical shift index (CSI) to detect changes in secondary structure of AcCYS_DL upon binding to papain. The CSI plots of both AcCYS_DL in the free and bound forms showed that the length of the α-helix of AcCYS_DL is shortened upon association with papain while the β-sheet structure remains unchanged (Figure 7A and 7B). In accord with the chemical shift perturbation results, we confirmed that the first turn of the α-helix (residue 56–58) of AcCYS_DL collapses upon association with papain. However, the role of the residues 86 and 88 to the interaction of AcCYS and papain remains unclear.

H/D exchange experiments have been widely applied to map the solvent accessible surface of proteins. The amide protons on the solvent accessible surface of proteins are labile to exchange with solvent deuterium. On the other hand, the most protected amide protons are located in the interior of protein or involved in hydrogen bonds of secondary structure. Hence we used H/D exchange experiments to map the interaction surface and secondary structure changes of AcCYS/papain complex. As shown in Figure 7C, the H/D exchange rates of amide protons of V90, and V91 of AcCYS_DL in the bound form were slower than those in the free form. The amide protons of V90 and V91 on the conserved loop were exposed to solvent in the free form, but protected from exchange with solvent once binding to papain. On the contrary, the H/D exchange rates of amide protons of E60, D61, and L62 of bound AcCYS_DL were much faster than those in the free form. Based on our NMR structure of free AcCYS, the amide protons of residue 60–62 are hydrogen bonded to their respective carbonyl acceptors of residue 56–58 in the helix and therefore protected from exchange with solvent. Rapid exchange of these amide protons with deuterium in the bound form indicated that the hydrogen bonds in the first turn of α-helix break up upon association with papain, further supporting the notion of partial helix collapse. The exchange rates of amide protons of residue 45 to 48 (GIYD) were too fast, hence unsuitable as probes in H/D experiments.

In combining the chemical shift perturbation, CSI, and H/D exchange studies, we proposed that the two conserved loop regions of AcCYS, including residues 45 to 48 (GIYD) and residues 89 to 91 (QVV) play primary roles in the recognition to papain, whereas the conserved W120 is not involved. The conformational rearrangement in the helical region of AcCYS is induced upon binding to papain.

### Molecular Model for the Interaction between AcCYS and Papain

In order to provide the structural insight for the recognition and inhibition of AcCYS to papain, structures of AcCYS/papain complex were simulated *in silico*. The initial structure model of AcCYS/papain complex was built from the crystal structure of taro cystatin/papain complex with the taro cystatin replaced by our NMR AcCYS structure. Rigid body docking simulation, ZDOCK was used to model the structure of AcCYS/papain complex. The structure with the best ZRank score was selected for further energy minimization. The energy-minimized dock structure of AcCYS/papain is shown in Figure 8A. The residues in the conserved motif 1 and 2 of AcCYS are in proximity to the active site of papain, whereas residue W120 of AcCYS in the third conserved motif protruded from the binding surface. The dock structure of AcCYS/papain was then simulated with restrained molecular dynamics simulation. The restraints include the hydrogen bonds derived from hydrogen exchange and CSI data, intramolecular restraints of hydrophobic clusters in AcCYS, and two intermolecular restraints derived from the dock structure (Table S2). Restrained molecular dynamics simulation was performed for 1 ns and the final simulated AcCYS/papain structure is shown in Figure 8B. The residues in the conserved motif 1 (G44–G45) and 2 (Q89–V91) of AcCYS completely blocked the active site of papain, including C25, H159, and N175. The first turn of the α-helix collapsed. An intermolecular H-bond was found between G45 amide proton of AcCYS and the side chain carboxyl of D158 of papain in the simulated complex structure; the carboxyl of D48 of AcCYS formed a salt bridge with K156 of papain (Figure 8C). In addition, the side chain of Y47 of AcCYS was found to be close to Y61, Y66, and W69 residues of papain and therefore may form a stable intermolecular aromatic cluster to stabilize the complex structure (Figure 8D). These data also explain why the chemical shifts of Y47 and D48 were significantly perturbed upon binding to papain.

Based on the data collected, we propose a molecular model to depict the events following the interaction between AcCYS and papain. Residues V90 and V91 in the second conserved loop (CM2) of AcCYS are responsible for initial contact with papain. Upon engagement, G44 and G45 of the first conserved motif (CM1) move toward the catalytic cleft of papain. This movement pulls down the α-helix, causing it to undergo partial unwinding. Intermolecular forces including salt bridge, H-bond and aromatic cluster between AcCYS and papain form and stabilize the complex structure. The finely tuned structural rearrangements allow AcCYS to tightly bind to papain and completely block the entry to its active sites.

### Conclusions

We have solved the solution structure of the pineapple cystatin by multi-nuclei NMR spectroscopy. A combination of techniques including chemical shift perturbation, CD spectroscopy, and computational modeling was employed to identify sites of contact, determine secondary structure rearrangements, and finally build a model for the interaction between pineapple cystatin and papain. Consistent with other homologous cystatins that bind to the target cysteine proteases by 3-point interactions, the conserved glycine in the N-terminal region and the second hairpin loop of pineapple cystatin make extensive contacts with papain. In contrast, the tryptophan residue in the third conserved loop does not appear to participate in the binding interaction. Overall, binding of pineapple cystatin to papain results in slight rearrangements of the secondary structures, including partial melting of the α-helix and subtle movement of the surface loop toward the active site cleft. These structural perturbations are in turn stabilized by a number of intermolecular forces formed between the two molecules. We also found a non-signal peptide N-terminal extension in pineapple cystatin that is distinctive among other plant species. While this unstructured N-terminal peptide does not seem to affect the overall fold and stability, it reduces the inhibition potency of pineapple cystatin by 30%, likely due to interference of interaction between the first conserved loop of the cystatin and the papain.

## Supporting Information

Figure S1 **2D ^1^H-^15^N-HSQC spectrum of 1.0 mM uniformly ^15^N-enriched AcCYS_DL in 20 mM KCl at pH 6.5, 303 K.** Assignments of the backbone amide protons and ^15^N cross peaks are indicated in the figure. The most crowded region is expanded at the top left corner of the figure for clarity purpose.
(TIF)

Table S1 **Backbone resonance assignments of free and papain bound forms of AcCYS_DL.**
(PDF)

Table S2 **Intra- and inter- molecular restraints used in the MD simulation of AcCYS_ DL/papain complex structure.**
(PDF)
