# Supplementary material for: Solution Structure of a Phytocystatin from Ananas comosus and Its Molecular Interaction with Papain
Source: PLoS One. 2012 Nov 6;7(11):e47865. doi: 10.1371/journal.pone.0047865 (PMC3490968; doi:10.1371/journal.pone.0047865)
Supplement: Table S1 — Backbone resonance assignments of free and papain bound forms of AcCYS_DL. (PDF) [file pone.0047865.s002.pdf]

Table S1. Backbone resonance assignments of free and papain bound forms of AcCYS_DL*

	Free AcCYS_DL							bound form AcCYS_DL							$\Delta\delta_{residue}$
	chemical shift (ppm)														
	N	NH	Hα	Hβ	Cα	Cβ	CO	N	NH	Hα	Hβ	Cα	Cβ	CO	
L29	121.414	8.093	4.475	1.554,1.684	55.434	42.430	176.883	121.444	8.094	4.299	1.504	ND	ND	176.907	0.0881
H30	120.037	8.592	4.702	3.225,3.102	55.334	29.250	174.514	120.300	8.593	4.712	3.239,3.124	ND	ND	174.440	0.0168
L31	124.315	8.304	4.305	1.926,0.887	55.375	42.026	177.264	124.227	8.365	4.321	1.590	55.312	42.082	177.291	0.0273
E32	120.740	8.533	4.251	2.063,1.912	56.316	31.154	176.226	120.817	8.553	4.263	2.064,1.911	56.703	29.590	176.263	0.1747
D33	120.652	8.222	4.579	2.710,2.636	54.284	41.006	176.034	120.858	8.234	4.568	2.721,2.624	54.422	40.872	176.039	0.0297
D34	120.477	8.216	4.565	2.678	54.276	41.052	176.028	120.461	8.222	4.569	2.684	54.355	40.642	176.221	0.0563
Q35	119.568	8.240	4.305	2.145,1.967	55.687	29.369	175.851	119.627	8.229	4.300	2.150,1.973	55.481	29.272	175.876	0.0297
E36	122.821	8.234	4.285	1.965,2.147	54.724	27.224	174.366	122.938	8.247	4.310	1.974	ND	ND	ND	0.0176
P37	NA	NA	4.839	2.336,2.076	62.458	33.698	176.074	ND	ND	4.839	2.363,2.109	62.124	33.790	176.074	0.0418
R38	122.323	8.433	4.319	1.789,1.735	55.497	29.988	176.542	122.264	8.440	4.321	1.766	55.643	30.800	176.206	0.0818
E39	120.711	8.281	4.317	1.618	56.662	30.953	175.779	121.033	8.305	4.372	1.493	56.316	30.052	175.854	0.1119
H40	120.154	8.351	4.614	3.029	53.787	28.894	172.600	120.413	8.363	4.706	3.142	ND	ND	ND	0.0556
P41	NA	NA	4.401	2.241,1.871	62.903	31.835	176.354	NA	NA	4.399	2.271,1.882	63.017	31.676	176.729	0.0587
I42	121.297	8.339	4.155	1.844	61.289	38.544	176.345	121.651	8.348	4.133	1.851	61.205	38.182	176.349	0.0510
M43	124.755	8.451	4.565	2.063	55.327	32.678	176.366	124.931	8.423	4.601	2.035	ND	ND	ND	0.0282
G44	110.953	8.527	4.100	NA	44.814	NA	174.530	110.376	8.493	3.983	NA	44.441	NA	174.654	0.0832
G45	108.375	8.339	3.840,4.073	NA	44.690	NA	173.383	112.185	8.186	3.817,4.264	NA	44.168	NA	176.151	0.3419
I46	120.858	8.005	4.305	1.502	61.308	38.400	175.949	121.412	8.367	4.072	1.635	62.383	38.271	176.070	0.2101

Y47	125.165	8.668	4.948	3.006	56.081	40.326	174.633	121.690	8.285	4.734	2.875,3.165	57.160	38.214	175.321	0.3467
D48	121.707	8.510	4.579	2.637	55.439	41.312	175.063	121.446	7.958	4.583	2.619	54.172	41.001	175.105	0.2634
A49	126.278	8.310	4.870	1.194	49.416	19.021	174.035	125.429	8.071	4.900	1.237	ND	ND	175.035	0.1787
P50	NA	NA	4.387	2.336,1.899	62.843	31.748	176.697	NA	NA	4.404	2.276,1.891	62.856	31.637	176.832	0.0325
L51	123.026	8.404	4.483	1.557,1.694	54.968	41.830	176.985	122.205	8.311	4.336	1.604	55.082	41.954	177.110	0.0804
N52	119.949	8.650	4.784	2.870,3.020	52.636	38.962	175.258	119.235	8.434	4.708	2.816	53.076	38.652	174.858	0.1226
N53	117.869	8.480	4.661	2.842	54.021	38.404	176.071	119.055	8.412	4.710	2.790	53.439	38.514	175.484	0.1031
E54	120.330	8.551	4.278	2.076	57.821	29.442	177.024	121.004	8.487	4.269	1.992,2.063	56.997	29.646	176.386	0.1135
N55	117.136	8.252	4.825	2.829,2.979	53.084	38.869	176.846	118.243	8.353	4.715	2.788	53.329	38.710	175.718	0.1426
G56	108.609	8.105	3.977	NA	46.537	NA	175.268	108.666	8.225	3.900	NA	45.535	NA	174.441	0.1482
F57	120.682	8.187	4.503	3.131,3.070	58.708	38.744	176.783	120.461	8.222	4.563	3.110,3.030	58.100	38.883	176.082	0.0994
D58	121.209	8.398	4.415	2.705	55.647	40.178	178.274	121.170	8.620	4.569	2.687	54.607	40.632	177.990	0.1599
K59	118.162	8.158	4.046	1.981,1.694	57.482	30.600	177.782	119.500	8.210	4.041	1.923,1.767	57.457	31.057	177.675	0.0699
E60	119.949	8.175	3.704	2.227,2.008	60.132	28.795	177.581	119.787	8.108	3.700	2.201,1.992	60.299	28.555	177.634	0.0482
D61	119.773	8.105	4.128	2.528	57.772	41.632	179.383	119.562	8.030	4.150	2.528	57.572	41.252	179.440	0.0628
L62	119.803	7.624	3.772	1.762,0.983	57.771	43.490	178.036	120.125	7.684	3.797	1.722	57.838	43.097	178.065	0.0589
A63	121.590	8.052	3.827	1.406	54.958	20.825	178.633	121.644	8.030	3.816	1.360	55.032	20.683	178.663	0.0280
R64	117.869	8.480	3.430	1.530,1.612	60.327	29.796	179.680	117.664	8.370	3.433	1.555	60.416	29.441	179.696	0.0658
F65	121.121	7.870	4.087	3.348,3.211	60.697	37.512	175.317	121.063	7.866	4.092	3.324,3.187	60.682	37.315	175.275	0.0309
A66	121.795	8.046	3.458	1.516	55.041	18.577	177.535	121.771	8.040	3.446	1.500	55.063	18.160	177.626	0.0539
V67	115.349	7.929	2.993	2.008	66.599	31.707	176.433	115.261	7.883	2.979	1.993	66.460	31.577	176.427	0.0339
R68	119.187	7.812	3.909	1.830	59.624	29.870	178.847	119.187	7.801	3.902	1.840	59.509	29.720	178.825	0.0287
E69	119.158	8.240	3.868	1.571	58.464	29.400	178.842	119.187	8.241	3.858	1.562	58.369	29.259	178.922	0.0280

Y70	121.737	8.105	3.458	2.117,1.757	62.342	37.046	178.232	121.446	8.067	3.458	2.137,1.759	62.369	37.026	178.366	0.0270
N71	120.301	8.627	4.100	2.842	55.564	37.187	178.810	120.298	8.620	4.080	2.853	55.486	37.028	178.883	0.0299
N72	117.312	8.187	4.442	2.850,2.758	55.426	38.360	177.592	117.312	8.188	4.447	2.835,2.773	55.363	38.185	177.644	0.0294
K73	118.367	7.900	4.059	1.680	57.954	32.638	177.495	118.367	7.883	4.061	1.685	57.891	32.676	177.532	0.0120
N74	113.151	7.554	4.688	2.487,1.584	52.831	39.186	173.499	113.163	7.540	4.688	2.489,1.567	52.937	38.979	173.548	0.0340
N75	119.363	7.730	4.415	3.020,2.692	54.244	36.774	173.524	119.363	7.719	4.415	3.026,2.685	53.977	36.484	173.542	0.0485
A76	120.594	8.052	4.702	1.488	49.911	21.849	175.846	120.623	8.042	4.709	1.494	50.026	21.973	175.889	0.0138
L77	120.711	8.369	4.551	1.817,1.379	53.176	42.533	177.645	120.714	8.367	4.559	1.826,1.384	53.200	42.282	177.665	0.0361
L78	121.151	8.639	4.346	2.022,0.900	56.157	42.542	177.974	121.160	8.637	4.368	2.030,0.900	56.176	42.300	178.000	0.0362
E79	120.008	8.404	4.743	2.145,2.049	54.105	32.158	176.178	119.956	8.399	4.745	2.140,2.050	53.860	32.105	176.349	0.0344
F80	126.044	9.307	4.059	2.993	60.662	39.523	173.869	125.967	9.309	4.067	2.988	60.682	39.487	173.886	0.0149
V81	127.451	7.882	3.840	1.502	64.455	32.765	174.308	127.509	7.889	3.830	1.484	64.386	32.673	174.344	0.0219
R82	110.719	6.985	4.333	1.885,1.639	55.896	30.718	173.921	110.719	6.992	4.326	1.842,1.661	55.805	31.077	174.051	0.0313
V83	121.854	9.225	4.360	2.186	62.681	32.155	174.701	121.942	9.273	4.323	2.188	62.756	32.014	175.222	0.0642
V84	125.985	9.073	3.963	1.776	63.766	32.961	175.731	127.393	9.095	3.926	1.759	63.996	32.652	175.829	0.0775
K85	116.755	7.618	4.497	1.871,1.803	55.931	35.693	172.957	116.843	7.619	4.551	1.903,1.761	55.629	35.390	173.207	0.0616
A86	125.077	9.096	5.481	1.447	51.551	22.047	175.108	124.608	8.728	5.436	1.420	51.535	22.510	175.425	0.1603
K87	119.598	8.938	5.085	1.789,1.598	54.644	35.809	175.949	120.789	8.859	4.888	1.813,1.916	55.073	35.942	175.398	0.1223
E88	123.524	8.996	5.276	2.022,1.519	54.844	33.083	174.188	123.202	8.857	5.450	2.000,1.607	55.096	32.820	175.232	0.1477
Q89	120.125	8.504	4.729	2.319,1.912	53.850	34.165	174.032	121.417	8.912	4.686	2.400,2.079	54.535	32.932	173.986	0.2329
V90	129.590	9.372	4.469	2.200	63.864	31.233	176.210	127.652	8.891	4.454	2.133	63.935	31.148	176.365	0.2133
V91	127.949	7.958	4.483	2.200	60.707	32.359	174.749	128.857	7.995	4.416	2.204	61.100	30.193	174.650	0.2399
S92	120.154	8.298	4.647	4.350,4.004	58.232	61.790	175.706	120.055	8.386	4.338	3.997,4.338	58.058	61.995	175.640	0.1330

G93	110.397	8.117	4.975,4.087	NA	44.930	NA	172.831	109.576	8.042	4.900,4.047	NA	44.706	NA	172.866	0.0616
M94	122.704	9.067	5.413	1.981,1.707	53.480	35.486	173.794	122.323	9.003	5.354	1.921,1.737	53.457	35.390	174.270	0.0653
M95	125.487	9.436	5.413	1.789,2.159	52.779	32.456	173.923	125.468	9.309	5.354	1.736, 2.144	52.744	32.380	173.795	0.0614
H96	124.960	9.249	5.003	3.034,2.897	55.200	32.593	174.731	125.468	9.309	4.900	3.081,2.873	55.368	32.406	174.637	0.0636
Y97	122.411	8.756	5.194	2.829	56.108	37.789	176.211	122.806	9.000	5.193	2.894	56.312	37.853	176.128	0.1034
L98	126.191	9.641	5.208	1.912,1.338	53.299	44.881	175.377	125.692	9.555	5.226	1.919,1.353	53.277	44.835	175.473	0.0450
T99	123.407	9.249	5.016	4.032	63.133	67.789	173.761	123.084	9.297	5.038	4.030	63.014	67.972	173.971	0.0363
V100	126.366	9.864	4.852	2.090	60.044	34.858	173.171	126.161	9.848	4.862	2.098	59.774	34.700	173.200	0.0399
E101	125.517	8.990	5.345	1.770,1.700	54.800	32.080	175.771	125.546	8.998	5.351	1.721	54.753	31.837	175.879	0.0373
V102	118.689	9.331	5.276	2.063	57.809	34.839	173.522	118.748	9.326	5.283	2.069	57.600	34.584	173.583	0.0427
N103	119.598	9.307	5.139	2.801,2.432	51.956	40.430	174.738	119.598	9.297	5.135	2.806,2.411	51.791	40.309	174.769	0.0287
D104	126.191	9.178	4.866	2.965,2.268	51.958	41.862	175.626	126.249	9.179	4.860	2.967,2.266	51.808	41.968	175.661	0.0161
A105	127.304	9.026	3.991	1.379	53.071	16.874	176.909	127.275	9.021	3.991	1.374	52.890	16.548	176.938	0.0474
G106	104.536	8.052	3.622,4.346	NA	45.250	NA	174.008	104.536	8.042	3.617,4.338	NA	45.138	NA	174.088	0.0151
K107	121.707	7.665	4.620	1.940,1.817	54.440	33.903	175.417	121.678	7.654	4.625	1.936,1.819	54.216	33.601	175.467	0.0475
K108	125.399	8.697	5.276	1.858,1.707	55.769	32.409	177.082	125.340	8.692	5.275	1.858,1.715	55.639	32.378	177.182	0.0217
K109	124.198	9.219	5.030	2.049,1.789	54.911	39.522	173.619	124.081	9.197	5.048	2.026,1.779	55.107	39.231	173.773	0.0490
L110	120.359	8.861	5.481	1.42,0.996	53.080	44.970	175.277	120.275	8.854	5.482	1.438,0.930	53.204	44.937	175.385	0.0220
Y111	121.414	8.979	4.989	1.912,1.338	55.849	43.310	174.090	121.356	8.974	4.982	1.902,1.326	55.643	43.317	174.195	0.0258
E112	120.418	9.172	5.235	1.981,1.830	54.422	31.703	174.991	120.492	9.166	5.219	1.931,1.852	54.546	31.787	174.982	0.0149
A113	130.645	9.706	5.317	1.393	49.628	21.757	175.077	130.383	9.721	5.347	1.392	49.617	21.722	175.322	0.0334
K114	124.843	8.797	5.413	1.735,1.625	54.800	35.338	175.254	124.462	8.734	5.419	1.737,1.618	54.837	34.985	175.556	0.0633
V115	127.421	9.295	4.893	1.981	60.926	35.844	173.004	127.099	9.267	4.897	1.997	60.933	35.485	173.128	0.0516

W116	130.674	9.149	5.823	3.458,2.924	54.241	32.192	174.183	130.732	9.150	5.825	3.426,2.951	54.169	31.795	174.346	0.0539
E117	128.271	9.624	5.016	2.200,1.625	54.390	33.957	175.028	128.066	9.525	4.998	2.207,1.615	54.138	33.116	174.440	0.1235
Q118	124.579	8.375	4.237	1.338	54.174	28.626	177.011	124.608	8.370	4.239	1.314	53.992	28.546	176.884	0.0293
V119	125.604	9.219	3.840	2.159	65.682	31.574	178.281	125.458	9.162	3.801	2.147	65.772	31.326	178.199	0.0474
W120	118.572	7.348	4.592	3.362	59.631	27.402	175.548	118.631	7.361	4.598	3.372	59.470	27.173	175.525	0.0380
M121	117.312	6.522	4.606	1.899,1.420	52.964	32.673	175.248	117.283	6.540	4.585	1.897,1.433	53.291	32.494	175.311	0.0458
N122	117.341	7.859	4.210	3.006,2.637	54.162	37.074	173.112	117.371	7.860	4.204	3.011,2.636	53.914	36.898	173.238	0.0401
F123	116.872	6.961	4.688	2.664,2.418	56.470	41.969	173.645	116.872	6.962	4.700	2.663,2.440	56.453	42.010	173.752	0.0135
R124	126.220	7.513	4.716	0.791,0.641	54.024	33.628	173.914	126.191	7.543	4.730	0.848,0.675	53.879	33.197	173.850	0.0582
Q125	119.978	8.856	4.661	2.131,2.418	54.682	31.783	175.024	120.000	8.860	4.657	2.236,1.867	54.551	31.761	174.987	0.0188
L126	130.645	9.706	4.675	2.104,1.106	55.624	42.647	175.097	130.483	9.693	4.687	2.113,1.127	55.399	42.596	175.342	0.0503
Q127	127.392	9.460	4.633	2.282,1.844	56.970	30.250	175.942	127.538	9.408	4.633	2.261,1.844	56.991	30.027	176.049	0.0497
E128	115.700	7.688	4.647	2.090,2.022	55.778	34.188	173.812	115.788	7.666	4.639	2.095,2.025	55.753	34.054	173.922	0.0284
F129	127.890	9.002	5.194	3.417,2.733	58.946	41.610	174.216	127.949	8.980	5.187	3.429,2.731	58.932	41.557	174.318	0.0212
T130	123.290	8.826	4.592	3.963	62.261	71.533	173.177	123.202	8.857	4.607	3.961	62.191	71.243	173.283	0.0443
Y131	129.267	8.949	3.266	2.815,2.664	58.274	37.415	174.244	129.267	8.939	3.279	2.798,2.653	58.173	37.495	174.320	0.0147
L132	128.242	8.386	4.182	1.160	54.362	43.241	176.709	128.300	8.376	4.173	1.149	54.281	43.058	176.677	0.0308
G133	104.800	6.111	3.348,4.004	NA	44.647	NA	170.929	104.801	6.108	3.357,4.018	NA	44.556	NA	170.893	0.0121
D134	120.154	8.351	4.907	2.938,2.541	54.790	40.909	175.343	120.219	8.351	4.899	2.938,2.545	54.783	40.872	175.356	0.0147
A135	129.326	7.776	4.303	1.382	53.053	19.000	181.997	129.326	7.772	4.108	1.152	ND	ND	181.944	0.0977

* A single quantity used as overall normalized chemical shift perturbation of each residue ($\Delta\delta_{residue}$) was expressed as described in the materials and methods.

$$\Delta\delta_{\text{residue}} = \left\{ \frac{1}{6} \left[(\Delta\delta_{H^N})^2 + (\Delta\delta_{H^\alpha})^2 + (\frac{\Delta\delta_N}{10})^2 + (\frac{\Delta\delta_{C^\alpha}}{4})^2 + (\frac{\Delta\delta_{C^\beta}}{4})^2 + (\frac{\Delta\delta_{C'}}{4})^2 \right] \right\}^{\frac{1}{2}}$$

where $\Delta\delta_{HN}$, $\Delta\delta_{H\alpha}$ $\Delta\delta_{N}$, $\Delta\delta_{C\alpha}$, $\Delta\delta_{C\beta}$ and $\Delta\delta_{C'}$ represents the chemical shift difference of AcCYS_DL upon association with papain for nucleus H^N, H^α, N, C^α, C^β and CO respectively.
