# Supplementary material for: Solution Structure of a Phytocystatin from Ananas comosus and Its Molecular Interaction with Papain
Source: PLoS One. 2012 Nov 6;7(11):e47865. doi: 10.1371/journal.pone.0047865 (PMC3490968; doi:10.1371/journal.pone.0047865)
Supplement: Table S2 — Intra- and inter- molecular restraints used in the MD simulation of AcCYS_ DL/papain complex structure. (PDF) [file pone.0047865.s003.pdf]

Table S2. Intra- and inter- molecular restraints used in the MD simulation of AcCYS_ DL/papain complex structure*

Distance restraints[1]						
Atom 1			Atom 2			
number	residue	atom name	number	residue	atom name	distance(Å)
47	TYR	HN	88	GLU	HN	3.03
49	ALA	HB1	86	ALA	HB2	2.52
62	LEU	HD22	98	LEU	HD11	3.62
62	LEU	HD22	98	LEU	HD12	2.57
62	LEU	HD22	98	LEU	HD21	4.54
62	LEU	HD11	115	VAL	HB	2.88
62	LEU	HD11	115	VAL	HG11	2.68
62	LEU	HD11	115	VAL	HG21	3.00
63	ALA	HB1	80	PHE	HD2	2.63
63	ALA	HB1	80	PHE	HE2	2.96
63	ALA	HB1	83	VAL	HG11	2.44
63	ALA	HB1	83	VAL	HG12	2.57
63	ALA	HB1	100	VAL	HG11	2.42
63	ALA	HB1	100	VAL	HG21	3.31
66	ALA	HB1	126	LEU	HD12	2.93
66	ALA	HB1	126	LEU	HD13	2.27
66	ALA	HB1	129	PHE	HD2	3.36
66	ALA	HB1	129	PHE	HE2	3.78
67	VAL	HG11	80	PHE	HN	2.94
67	VAL	HG21	80	PHE	HB2	2.27
67	VAL	HG23	129	PHE	HE2	2.24
70	TYR	HH	78	LEU	HD11	3.54
70	TYR	HH	78	LEU	HD21	2.61

Hydrogen bond restraints[2]						
Atom 1			Atom 2			distance(Å)
59	LYS	O	63	ALA	HN	2.38
60	GLU	O	64	ARG	HN	1.88
61	ASP	O	65	PHE	HN	2.12
62	LEU	O	66	ALA	HN	1.95
63	ALA	O	67	VAL	HN	2.05
64	ARG	O	68	ARG	HN	1.81
65	PHE	O	69	GLU	HN	1.89
66	ALA	O	70	TYR	HN	2.22

67	VAL	O	71	ASN	HN	1.9
68	ARG	O	72	ASN	HN	1.89
79	GLU	O	103	ASN	HN	1.92
79	GLU	HN	103	ASN	O	2.28
81	VAL	HN	101	GLU	O	1.89
82	ARG	O	101	GLU	HN	1.93
87	LYS	O	97	TYR	HN	1.92
89	GLN	O	95	MET	HN	1.97
89	GLN	HN	95	MET	O	2.63
84	VAL	HN	99	THR	O	1.98
85	LYS	O	99	THR	HN	2.02
96	HIS	O	115	VAL	HN	1.93
96	HIS	HN	115	VAL	O	1.91
98	LEU	O	113	ALA	HN	1.92
98	LEU	HN	113	ALA	O	2.03
100	VAL	O	111	TYR	HN	1.99
100	VAL	HN	111	TYR	O	1.92
102	VAL	O	109	LYS	HN	1.94
102	VAL	O	109	LYS	HN	1.97
104	ASP	HN	107	LYS	O	2.04
110	LEU	O	132	LEU	HN	1.99
112	GLU	HN	130	THR	O	1.99
112	GLU	O	130	THR	HN	1.94
114	LYS	O	127	GLN	HN	2.02
114	LYS	HN	128	GLU	O	2.15
116	TRP	HN	125	GLN	O	2.17
116	TRP	O	125	GLN	HN	2.03
118	GLN	HN	123	PHE	O	2.02

Intermolecular restraints[3]

Atom 1 (papain)			Atom 2 (AcCYS)			distance(Å)
159	HIS	HD1	90	VAL	HG11	1.50
177	TRP	HE1	91	VAL	HA	3.55

[*]Intramolecular and intermolecular distance restraints used in the MD simulation of AcCYS_ DL/papain complex. These restraints were derived from hydrogen exchange and CSI plot, H/D exchange, and chemical shift perturbation in the NMR experiments.
[1]Intramolecular distance restraints of hydrophobic clusters in AcCYS
[2]Hydrogen bond distance restraints derived from the hydrogen exchange and CSI data.
[3]Intermolecular distance restraints between AcCYS and papain, derived from the dock structure of AcCYS/papain
